# Supplementary material for: High-throughput viral enumeration of aquatic ecosystems via flow cytometry
Source: Appl Environ Microbiol. 2026 Jul 6;92(7):e01926-25. doi: 10.1128/aem.01926-25 (PMC13390381; doi:10.1128/aem.01926-25)
Supplement: Supplemental material — Tables S1 to S4; Fig. S1 to S6. [file aem.01926-25-s0001.pdf]

Supplemental Materials

High Throughput Viral Enumeration in Aquatic Ecosystems via Flow Cytometry

Madeline Bellanger-Perry, Pieter Visscher, Richard Allen White III

Supplemental Table 1. Average VLP concentrations at each location for each experiment type. Experiments ending in "-C" are chloroform treated samples. Experiments beginning with "B-" are benzonase treated samples. Averages and standard deviations are represented as VLPs mL^{-1}. Statistical comparisons of averages obtained using EFM are also presented.

Sample	Average	Std Dev	CV	EFM VLP mL^{-1} Avg	P-Value
FGL	1.82E+07	5.29E+06	29.10%	9.95E+05	N/A
GSL	1.16E+07	4.25E+06	36.82%	1.43E+06	N/A
WBS	4.47E+06	1.73E+06	38.74%	N/A	N/A
FGL-C	2.77E+06	1.94E+05	7.01%	9.95E+05	N/A
GSL-C	1.01E+07	1.51E+06	14.90%	1.43E+06	N/A
WBS-C	2.37E+07	1.85E+06	7.82%	N/A	N/A
B-FGL-C	3.28E+06	4.02E+05	12.24%	9.95E+05	7.8E-11
B-GSL-C	1.22E+07	1.75E+06	14.41%	1.43E+06	7.8E-11
B-WBS-C	2.13E+07	2.13E+06	10.03%	N/A	N/A

Supplemental Table 2. Results from the statistical analyses to compare treatment methodologies and identify degradation in FGL samples.

Comparison	Significant?	P Value
FGL - FGL.C	Y	2.00E-16
FGL - B.FGL.C	Y	2.00E-16
FGL.C - B.FGL.C	Y	8.70E-12
FGL All Rows	N	0.1301
FGL.C All Rows	Y	0.00119
FGL.C Row A - FGL.C Row B	N	0.9955775
FGL.C Row A - FGL.C Row C	N	0.9925509
FGL.C Row A - FGL.C Row D	N	0.3828107
FGL.C Row A - FGL.C Row E	N	0.7470396
FGL.C Row A - FGL.C Row F	N	0.9986493
FGL.C Row A - FGL.C Row G	N	0.9570131
FGL.C Row A - FGL.C Row H	Y	0.0018673
B.FGL.C All Rows	Y	2.00E-16
B.FGL.C Row A - B.FGL.C Row B	Y	0.000063
B.FGL.C Row A - B.FGL.C Row C	Y	0.0001995
B.FGL.C Row A - B.FGL.C Row D	Y	0
B.FGL.C Row A - B.FGL.C Row E	Y	0
B.FGL.C Row A - B.FGL.C Row F	Y	0
B.FGL.C Row A - B.FGL.C Row G	Y	0
B.FGL.C Row A - B.FGL.C Row H	Y	0

Supplemental Table 3. Results from the statistical analyses to compare treatment methodologies and identify degradation in GSL samples.

Comparison	Significant?	P Value
GSL - GSL.C	N	0.084
GSL - B.GSL.C	N	0.084
GSL.C - B.GSL.C	Y	1.10E-08
GSL All Rows	N	0.674
GSL.C All Rows	Y	8.18E-13
GSL.C Row A - GSL.C Row B	N	0.8433789
GSL.C Row A - GSL.C Row C	Y	0.0024881
GSL.C Row A - GSL.C Row D	Y	0.0113403
GSL.C Row A - GSL.C Row E	Y	0.0003011
GSL.C Row A - GSL.C Row F	Y	0.0000063
GSL.C Row A - GSL.C Row G	Y	0.000011
GSL.C Row A - GSL.C Row H	Y	0
B.GSL.C All Rows	Y	2.41E-07
B.GSL.C Row A - B.GSL.C Row B	N	0.01417
B.GSL.C Row A - B.GSL.C Row C	N	0.93723
B.GSL.C Row A - B.GSL.C Row D	N	0.23039
B.GSL.C Row A - B.GSL.C Row E	N	0.09091
B.GSL.C Row A - B.GSL.C Row F	N	0.05594
B.GSL.C Row A - B.GSL.C Row G	N	0.06061
B.GSL.C Row A - B.GSL.C Row H	N	0.05594

Supplemental Table 4. Results from the statistical analyses to compare treatment methodologies and identify degradation in WBS samples.

Comparison	Significant ?	P Value
WBS - WBS.C	Y	2.00E-16
WBS - B.WBS.C	Y	2.00E-16
WBS.C - B.WBS.C	Y	1.80E-08
WBS All Rows	Y	4.25E-06
WBS Row A - WBS Row B	N	0.21318
WBS Row A - WBS Row C	N	0.05195
WBS Row A - WBS Row D	N	0.10926
WBS Row A - WBS Row E	N	0.25152
WBS Row A - WBS Row F	Y	0.02797
WBS Row A - WBS Row G	Y	0.00673
WBS Row A - WBS Row H	Y	0.0016
WBS.C All Rows	Y	0.002428
WBS.C Row A - WBS.C Row B	Y	0.048
WBS.C Row A - WBS.C Row C	Y	0.03
WBS.C Row A - WBS.C Row D	N	0.101
WBS.C Row A - WBS.C Row E	Y	0.03
WBS.C Row A - WBS.C Row F	Y	0.036
WBS.C Row A - WBS.C Row G	Y	0.03
WBS.C Row A - WBS.C Row H	Y	0.03
B.WBS.C All Rows	Y	9.59E-08
B.WBS.C Row A - B.WBS.C Row B	Y	0.00203
B.WBS.C Row A - B.WBS.C Row C	Y	0.00758
B.WBS.C Row A - B.WBS.C Row D	N	0.22037
B.WBS.C Row A - B.WBS.C Row E	N	0.61055
B.WBS.C Row A - B.WBS.C Row F	N	0.68891
B.WBS.C Row A - B.WBS.C Row G	N	0.09091
B.WBS.C Row A - B.WBS.C Row H	N	0.26107

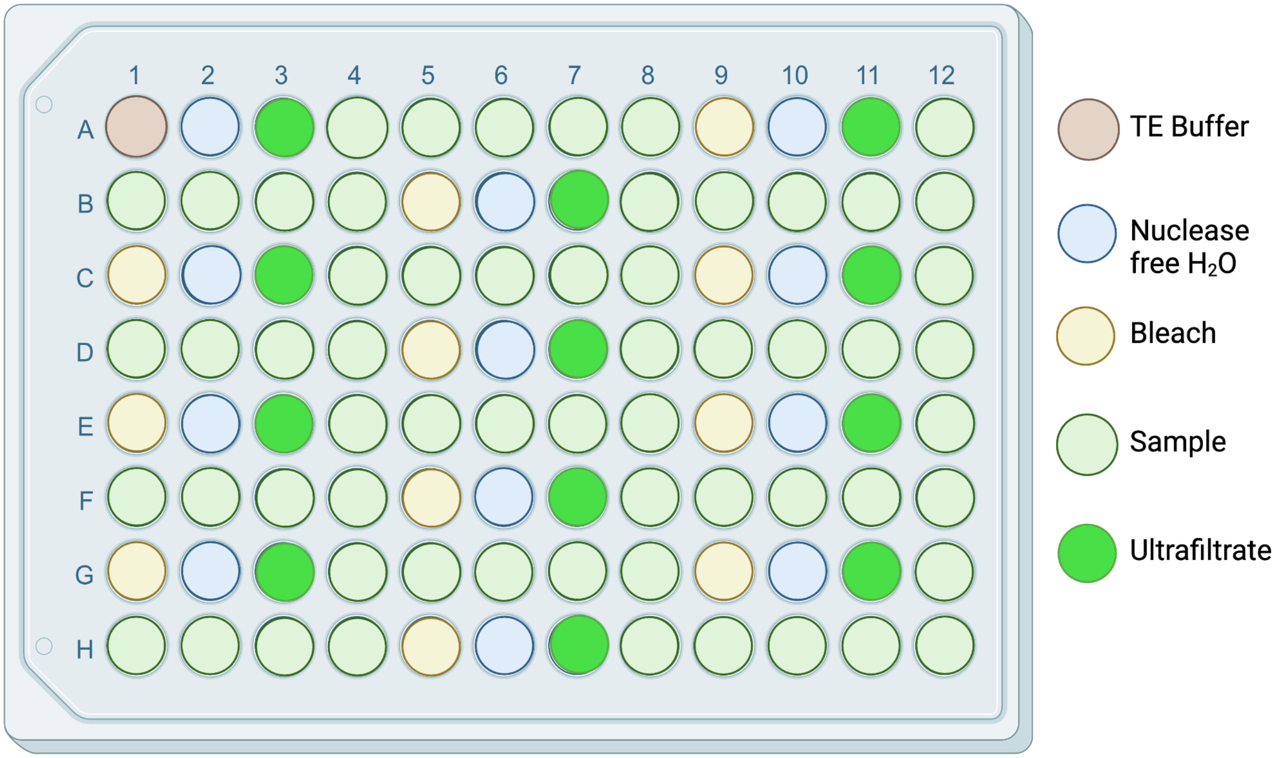

Supplemental Figure 1. Visual representation of the well plate plan. Well A1 is filled with TE buffer as a blank to determine the machine's base noise level. Twelve wells are filled with nuclease free water to rinse the cytometer and manage noise levels of the machine. Twelve wells are filled with ultrafiltrate that was treated the same as the sample to determine the base noise level of the sample. Eleven wells are filled with a 10% bleach solution to cleanse the machine throughout the run. Sixty wells are filled with the diluted sample. Throughout the well plate, a set of three wells containing 10% bleach, nuclease free water, and ultrafiltrate occur for every five sample wells. This ensures that an overestimation of VLPs are not being counted and the noise levels can be constantly managed.

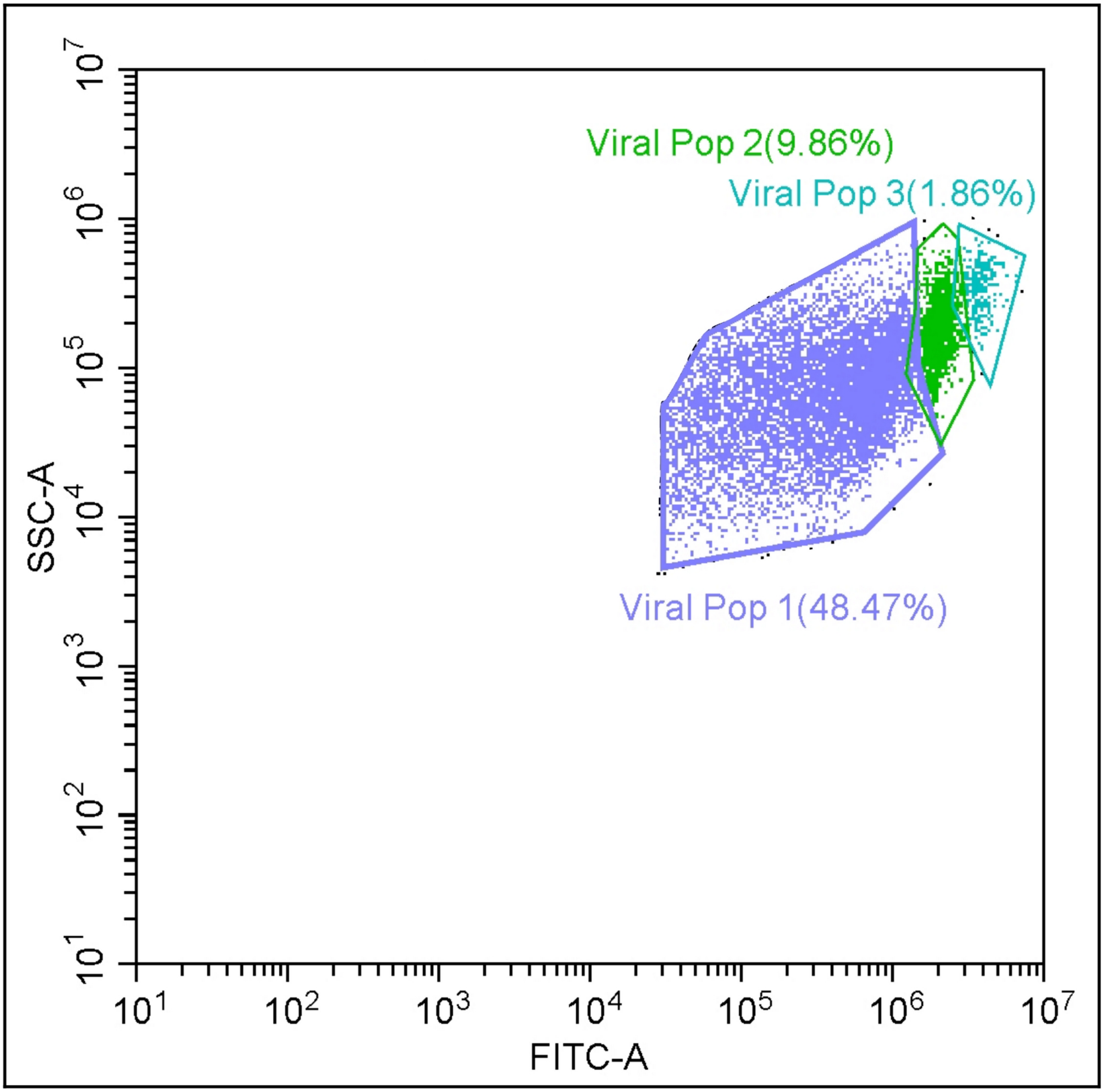

Supplemental Figure 2. Three distinct viral populations in a sample after treatment with chloroform and benzonase. Populations are identified and gated by looking for clusters of events (shown as dots) in the region of interest. Here, viral populations should have a high fluorescent intensity (FITC-A, X axis) and medium-high side scatter (SSC-A, Y axis). In this plot, only the gates of interest are shown.

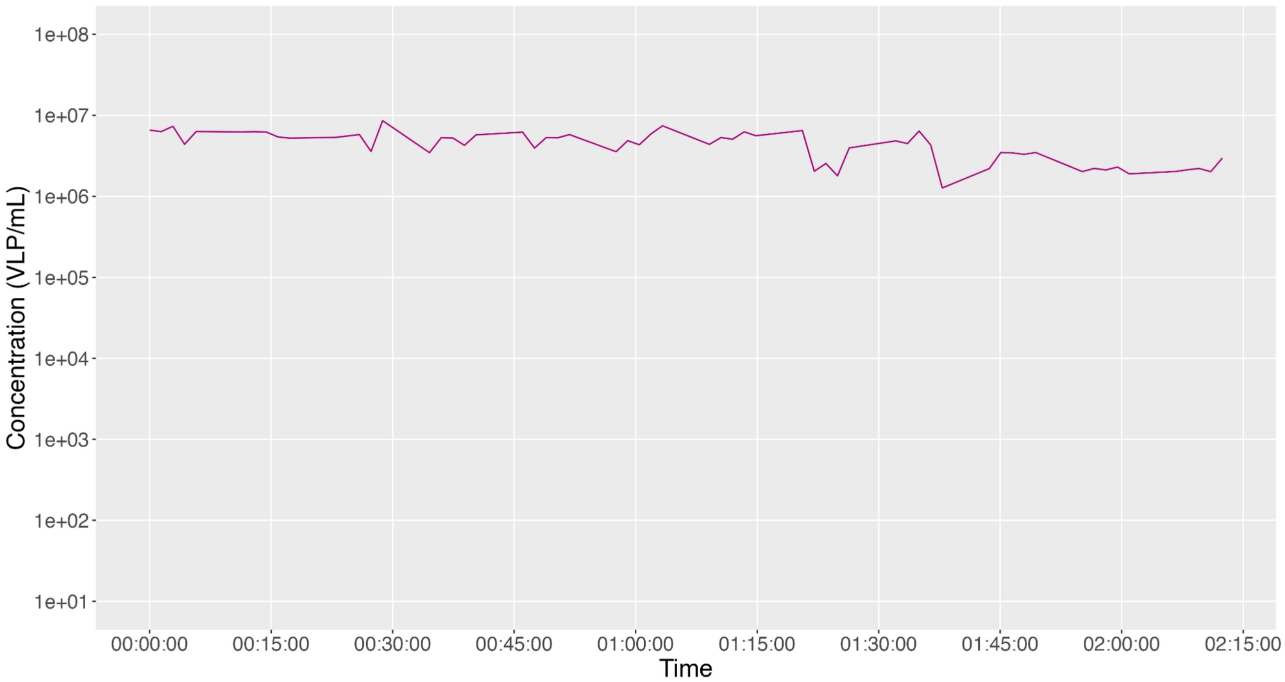

Supplemental Figure 3. Untreated WBS sample's VLP mL^{-1} over the course of the well plate's run on the flow cytometer. Time points are plotted for each well in reference to the time elapsed between that well and the first sample well, when T=0 hours, 0 minutes, 0 seconds (00:00:00). Two points of significant degradation can be seen between 1:15:00 and 1:30:00 and between 1:30:00 and 1:45:00.

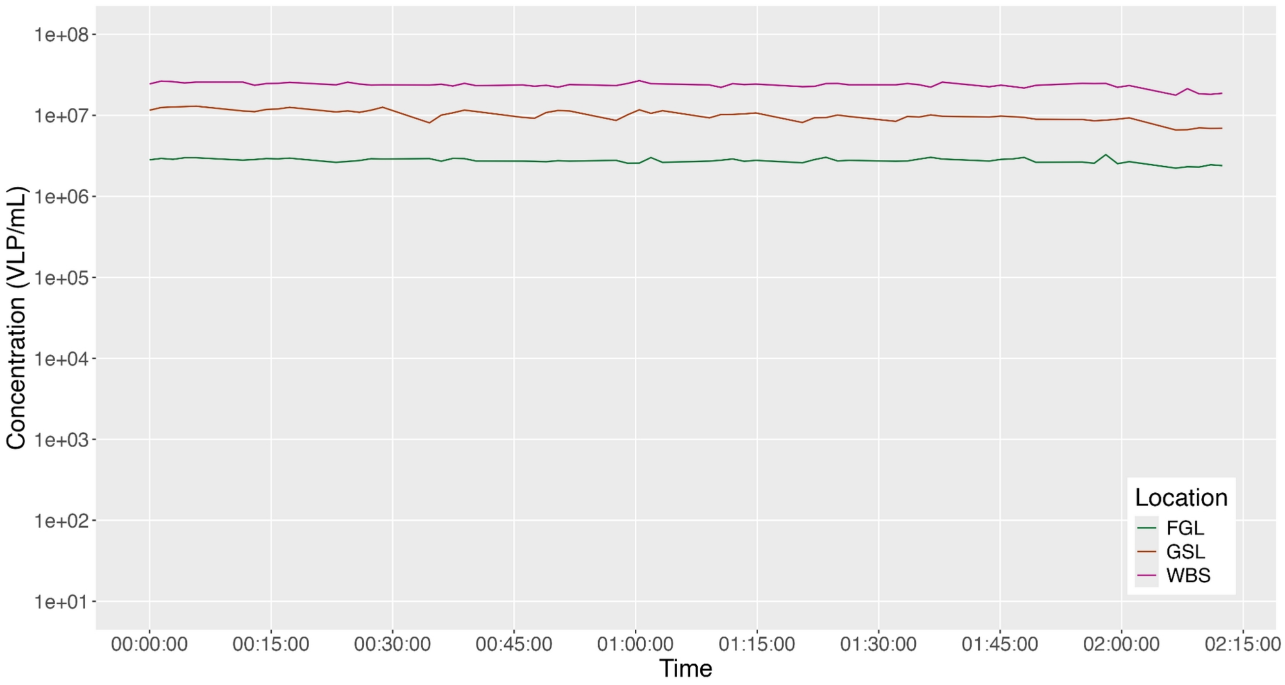

Supplemental Figure 4. Each sample location's VLP mL^{-1} over the course of the well plate's run on the flow cytometer. Samples were treated with chloroform. FGL is shown in green (bottom). GSL is shown in orange (middle). WBS is shown in pink (top). Time points are plotted for each well in reference to the time elapsed between that well and the first sample well, when T=0 hours, 0 minutes, 0 seconds (00:00:00).

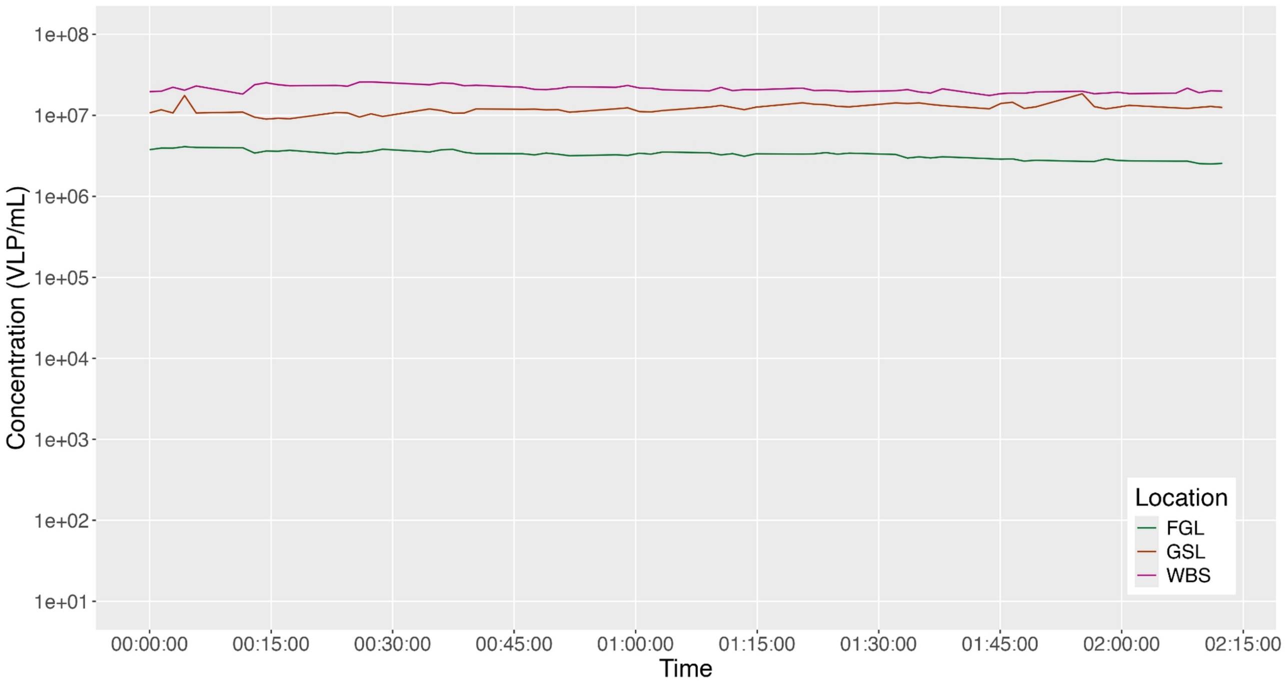

Supplemental Figure 5. Each sample location's VLP mL^{-1} over the course of the well plate's run on the flow cytometer. Samples were treated with chloroform and benzonase. FGL is shown in green (bottom). GSL is shown in orange (middle). WBS is shown in pink (top). Time points are plotted for each well in reference to the time elapsed between that well and the first sample well, when T=0 hours, 0 minutes, 0 seconds (00:00:00)

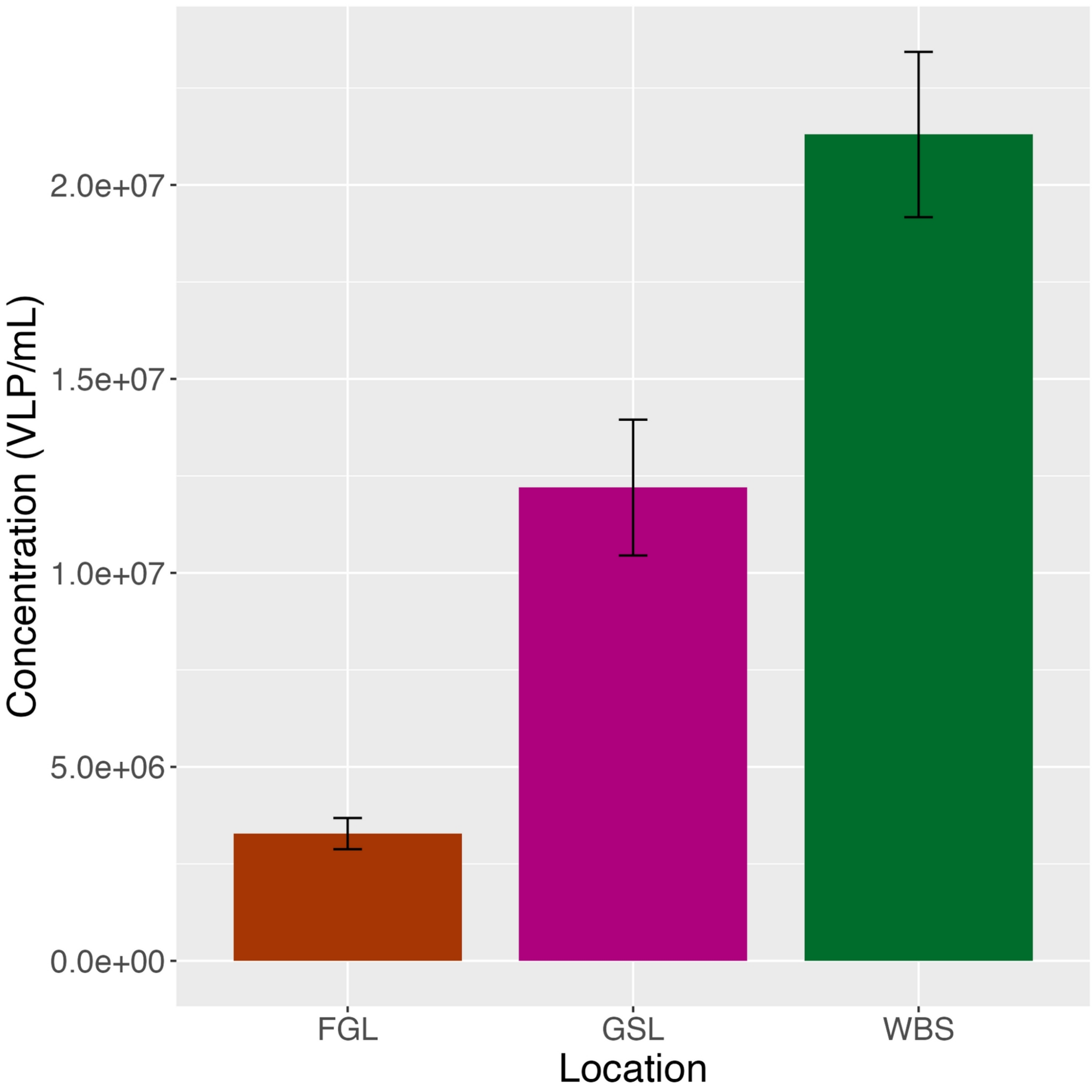

Supplemental Figure 6. Average VLP mL^{-1} at each sample location after chloroform and benzonase treatment. Error bars represent standard deviation values.
